# How do education and experience with mental illness interact with causal beliefs, eligible treatments and stigmatising attitudes towards schizophrenia? A comparison between mental health professionals, psychology students, relatives and patients

**DOI:** 10.1186/s12888-020-02580-6

**Published:** 2020-04-15

**Authors:** Stefania Mannarini, Alessandro Rossi, Cristina Munari

**Affiliations:** 1 Department of Philosophy, Sociology, Education, and Applied Psychology, Section of Applied Psychology, University of Padova, Padova, Italy; 2 Interdepartmental Center for Family Research, University of Padova, Padova, Italy

**Keywords:** Stigma, Mental illness stigma, Mental health, Schizophrenia, Latent class analysis

## Abstract

**Background:**

The main purpose of this study was to investigate the perception of schizophrenia in different categories of persons (directly and/or indirectly) involved with it. Hypotheses were made concerning the definition of a multi-class structure where each class should identify a profile characteristic of each respondent’s specific role, (e.g.: mental health professional, relative, patient, and student) and specific indicators of schizophrenia (e.g.: causal beliefs, eligible treatments, social distance, perceived dangerousness and public avoidance).

**Methods:**

This study involved 577 participants all in contact with schizophrenia with different roles. A Latent Class Analysis (LCA) was applied to define a latent structure of schizophrenia aspects. Such structure was expected be affected by the interaction between respondents’ roles, as external variable, and schizophrenia indicators as manifest variables.

**Results:**

A four-latent-class structure representing the four respondents’ roles was evidenced, further each class was characterized by schizophrenia indicators representing a profile for each role. Analogies and differences of views and preferences of the respondents’ roles concerning schizophrenia emerged clearly.

**Conclusions:**

The four groups of people involved with schizophrenia with different roles demonstrated to interact significantly with specific indicators of schizophrenia shedding new lights on the understanding of schizophrenia in its complexity.

## Background

Our knowledge and understanding of schizophrenia have improved through advances in psychiatric, neurological, medical, clinical and psychosocial studies, but the experts’ discussion about causal beliefs, possible treatments and specific indicators of schizophrenia is still open. Van Os and colleagues (2010) argue that although heritability is often emphasised in reference to the onset of schizophrenia, environmental factors also play an important role, suggesting that experiencing early life adversity might have a negative impact on the development of the ‘social brain’ during fundamental periods of life growth [1]. Insel (2010) points out that, after a century of studying schizophrenia, the origin of the disorder remains unknown and underlines that treatments have scarcely produced improved results for individuals with schizophrenia [2]. He argues that such outcomes might change as we approach schizophrenia as a neurodevelopmental disorder with psychosis as a late, predictable stage of the illness. As far as people’s thinking about schizophrenia is concerned, this form of mental illness represents a complex and multidimensional phenomenon in which both biological and environmental factors play an important role in the causality of schizophrenia. As a consequence, the possibility of developing treatment interventions is based either on medical approaches or psychological ones, or on an integrated perspective. Studies on people’s perception of schizophrenia, direct experience of manifestations and stigmatisation of such an illness, are frequently found in past and recent literature [3–11]. Stigmatising thinking concerning schizophrenia, such as prejudice, feeling in danger, public avoidance and various forms of discrimination, are still very frequent and represent a significant social problem [12–15]. A large part of society thinks that persons with schizophrenia are likely to be violent [5, 16], dangerous [17] and capable of hurting themselves or others [18]. They are perceived as less responsible for their behaviour than people with other ailments [19, 20]. At the same time, schizophrenia is perceived as more controllable than cancer or depression [21–23], and therefore people with schizophrenia are perceived as more responsible for their illness than someone who is physically sick or ill with depression [17]. Another stereotype about schizophrenia is that it is a stable disease, namely, one from which it is impossible to heal, and the best treatment plan is to alleviate its symptoms. Therefore, it is expected for the patient to stay passive and be obedient to mental health experts, who assumedly know more about the person’s situation than he/she does [22]. Researches as regards the effects of causal beliefs on stigmatisation, in particular, in reference to schizophrenia, have been carried out in different countries [4, 6, 11, 17, 20, 24–30]. Different groups of people that are involved with schizophrenia from different perspectives also have been studied and their views and preferences compared. Recently, Tarakita et al. (2019) carried out a study in Japan to determine whether etiological beliefs and their consequences in terms of attitudes towards mental illness are different among schizophrenia patients, their families and medical staff [31]. The results indicated that differences exist, in addition to some analogies, among the three groups. Other studies in the past and recent years have taken into consideration the relation between schizophrenia and different people’s perceptions. Among others, Magliano and colleagues (2004) studied beliefs about schizophrenia comparing mental health professionals with patients’ relatives [32]; Ribè and colleagues (2018) investigated quality of life in family caregivers of schizophrenic patients, analysing family functioning and professional support [33, 34]; and Amsalem and colleagues (2018) studied what patients with schizophrenia and their families know about such pathology [35].

In this study to advance the understanding of schizophrenia complexity and to further contribute to the discussion on mental health stigmatisation problems, analyses were aimed at combining specific indicators of schizophrenia, with groups of persons who are involved with schizophrenia having different roles. A question was posed: ‘How do education and direct experience with mental illness interact with causal beliefs, treatment and stigmatising attitudes towards schizophrenia?’ As far as specific aspects of schizophrenia are concerned, when considering causal beliefs, biogenetic and psychosocial causal attributions were expected to be the two extremes of a dimension, as found in previous studies [6, 36, 37]. Hypotheses were also made concerning relations between schizophrenia origins and possible treatments and of relations with a desire for social distance, perception of danger and general public avoidance of schizophrenic patients [3, 38–40]. Furthermore, a hypothesis was formulated that a latent structure, combining schizophrenia causal beliefs, social distance, perceived dangerousness and avoidance, should exist, also including possible schizophrenia treatments [4, 11]. Moreover, such a structure should take into account the thinking and preferences concerning schizophrenia of different groups of people; in other words, different profiles should emerge representing the beliefs and thinking of mental health professionals, relatives, patients and students. The expected latent structure was expected to be characterised by a latent variable linking the respondents’ roles with the variables, which are indicators of schizophrenia, and of possible stigmatising thoughts. A latent class analysis (LCA) was applied to assess such a structure, as explained below (see Analysis section).

## Methods

### Participants

A total of 600 participants were contacted (244 males [42.0%] and 333 females [58.0%], aged from 18 to 83 [*mean* = 40.63, *SD* = 16.31]) living in Northern Italy (see Table 1) and matched with inclusion/exclusion criteria. The inclusion criteria consisted of the following: being a native Italian-speaker, being over 18 years old, having some kind of knowledge or experience with people with mental illness (e.g.: work in a mental health service, relatives, psychology students etc.), who had experienced the onset and/or an acute episode of a clinical mental health disorder during the last month. Exclusion criteria consisted of the following: illiteracy or inability to complete the assessment procedure due to vision and/or cognitive impairments. The participants belonged to four different groups with an increasing experience of interaction with mental illness: psychology students, mental health professionals, psychiatric patients’ relatives and psychiatric patients. Out of the total participants, 23 individuals (3.8%) did not complete the procedure, more precisely, eight patients, four relatives, six mental health professionals and five psychology students. Thus, the overall group of participants consisted of 577 individuals.

**Table 1:** Participants descriptive statistics

	Overall (*n* = 577)		Patient (*n* = 157; 27.2%)		Family (*n* = 135; 23.4%)		MH professionals (*n* = 102; 17.7%)		Students (*n* = 183; 31.7%)	
Age (*M, SD*)	40.63	16.31	44.87	12.66	53.76	14.94	45.88	12.40	24.30	4.20
Gender (*n*, %)										
Male	244	42.0%	87	55.4%	51	37.8%	31	30.4%	75	41.0%
Female	333	58.0%	70	44.6%	84	62.2%	71	69.6%	108	59.0%

Psychiatric patients, their relatives and mental health professionals were enrolled in local territorial facilities, whereas university students were recruited at the university of Padua. Each participant was recruited individually. As far as the relatives group was concerned, each participant was asked if he/she also undertook or had undertaken a psychological or psychiatric treatment.

After written informed consent was obtained from each participant and data anonymity for research use only was guaranteed, the vignette that was the basis of the research survey was individually administered.

The research project conformed to the Declaration of Helsinki norms, and it was previously approved by the Ethics Committee of the University of Padua (protocol: 1734).

### Instruments

A number of instruments for measuring attitudes towards mental illness and causal beliefs and treatments exist in the Italian language, such as Community Attitudes to the Mentally Ill (CAMI) [41], the Questionnaire on the Opinions about Mental Illness [42], the Attribution Questionnaire (AQ-27) [43, 44], the Mental Disorders Causal Beliefs (MDCB) scale [36], and the Mental Disorder Therapy Relationship scale [45]. Such instruments are self-report questionnaires that consider one or several aspects characterising the multi-factor structure of mental illness, and they mostly refer to mental illness, in general ignoring the particularity of the different mental disorders [46]. In order to overcome such a limitation, in this study, a vignette approach was used to examine the validity of the expected latent structure characterised by schizophrenia indicators, namely, social distance, dangerousness and avoidance as perceived by four different groups of people, together with causal beliefs and recommended treatments [46]. In line with the large amount of previous scientific literature using the vignette approach [4, 11, 20, 47, 48] in this research, vignettes cognitively tested in the Italian environment in the studies by Mannarini, Boffo, Rossi and Balottin (2018) and Mannarini and Boffo (2015) were applied. In order to make sure that each vignette was properly understood, the participants of the patients group in particular were individually assisted during the vignette administration. A vignette is a story and/or a brief sketch about a person presenting the most common problems and symptoms typical of schizophrenia, which was developed according to DSM-5 diagnostic criteria [49], and the mental health disorder was not mentioned in any part of the text [4, 11]. Moreover, the gender of the vignette protagonist was counterbalanced across participants. Participants were requested to evaluate the possible origin of the problems of the protagonist, the possible treatments to cure his/her problems, the desire for social distance from that person, the degree of social dangerousness of the described person and the desire of people in general to avoid that person [46].

#### Causal belief

The respondents indicated their agreement on 10 items regarding 10 possible causes of the problems of the person described in the vignette. Five items are related to biogenetic causes (i.e., heredity, biogenetic predisposition and brain injury), and the other five items refer to psychosocial causes (i.e., traumatic childhood experiences, deprived family environment and stress). To compute the total score, the items referring to psychosocial causes have to be reversed. The sum of the 10 items gives the final score. Higher scores indicate a higher tendency to attribute etiology to biogenetic factors. Cronbach’s alpha for this scale was equal to 0.84.

#### Treatment

The respondents were asked to evaluate seven possible treatment approaches to cure the problems of the vignettes’ protagonist. Three items suggested psychiatric treatments (i.e., surgical operation, hospitalisation and drug prescription) and four items covered more psychological therapeutic approaches (i.e., family therapy, group therapy and individual psychotherapy). Even in this case, to compute the total score, the items referring to psychological approaches have to be reversed. The sum of the seven items gives the final score. Higher scores indicated a higher tendency to recommend medical treatments. Cronbach’s alpha for this scale was equal to 0.73.

#### Distance

Social distance from persons with schizophrenia was evaluated with five items that investigated the respondent’s preference not to be involved personally with the person described in the vignette. Cronbach’s alpha for this scale was equal to 0.88.

#### Dangerousness

Dangerousness was evaluated with five items related to the possibility of the protagonist exhibiting violent behaviour towards others. Cronbach’s alpha for this scale was equal to 0.87.

#### Avoidance

The tendency of people to avoid the person described in the vignette was assessed with four items asking an opinion about what people would generally do when involved with a mentally ill person. Cronbach’s alpha for this scale was equal to 0.73.

Items were rated on a Likert-type scale ranging from 1 (completely disagree) to 4 (completely agree). The items of both the psychosocial causal beliefs and the psychological treatment approach were score-reversed. Higher scores for social distance, dangerousness and avoidance indicated high degrees of distance, dangerousness and avoidance, respectively.

### Data analysis

Data were elaborated by means of LCA (e.g.: [50]) This methodological approach was chosen in order to identify a structure that might represent the relations between the observed variables characteristic of this study at a latent level.

According to the LCA assumptions, latent variables are categorical and are structured in a number of classes, which describe the presence or absence of specific peculiarities for each variable. In this study, as regards observed variables, such as roles of the participants, causal beliefs, recommended treatments, social distance, dangerousness and avoidance, a hypothesis was made that a structure of four classes typical of a latent variable X should emerge where each class should identify a profile [51, 52] characteristic of a specific role, either professional, or family, or patient, or student, and of the observed variable indicators for schizophrenia. The observed variables were expected to be related in different ways to the participants’ roles. In the psychological and medical literature, LCA has been largely applied in past and recent years [53–61]. Once the latent structure is identified, LCA allows entering other variables in the model as covariates that might influence the latent structure; these are called external variables. In this study, the variable indicators of schizophrenia were first introduced as manifest variables, namely causal beliefs, eligible treatment, social distance, dangerousness and avoidance. Then the participant roles were introduced successively as external variables in order to see if different roles might affect the organisation of the manifest variables’ latent structure. In order to satisfy the main assumption of the LCA, all variables were categorised, except for the variable role that was already categorical [62–66]. In particular as regards the two variables of causal belief and eligible treatments, the participants’ total score distributions were divided into three percentile categories as follows [62–64]: below the 33rd percentile (category 1: psychosocial beliefs/psychological treatment approach), between the 33rd and the 66th percentile (category 2: bio-psychosocial belief/integrated medical and psychological treatment) and above the 66th percentile (category 3: biological belief/medical treatment). The medium category 2 was considered important in order to verify mixed preferences concerning the origin of schizophrenia, namely hereditary but also environmental, and preferences for an integrated treatment both medical and psychological. As far as the other variables are concerned, these consist of the following: social distance, dangerousness and avoidance. In order to demonstrate, in particular, the respondents’ preferences at the upper and lower levels of the dimensions, their distributions were discretised into two categories, below and above the median and designated low and high, respectively.

**Fig. 1:**
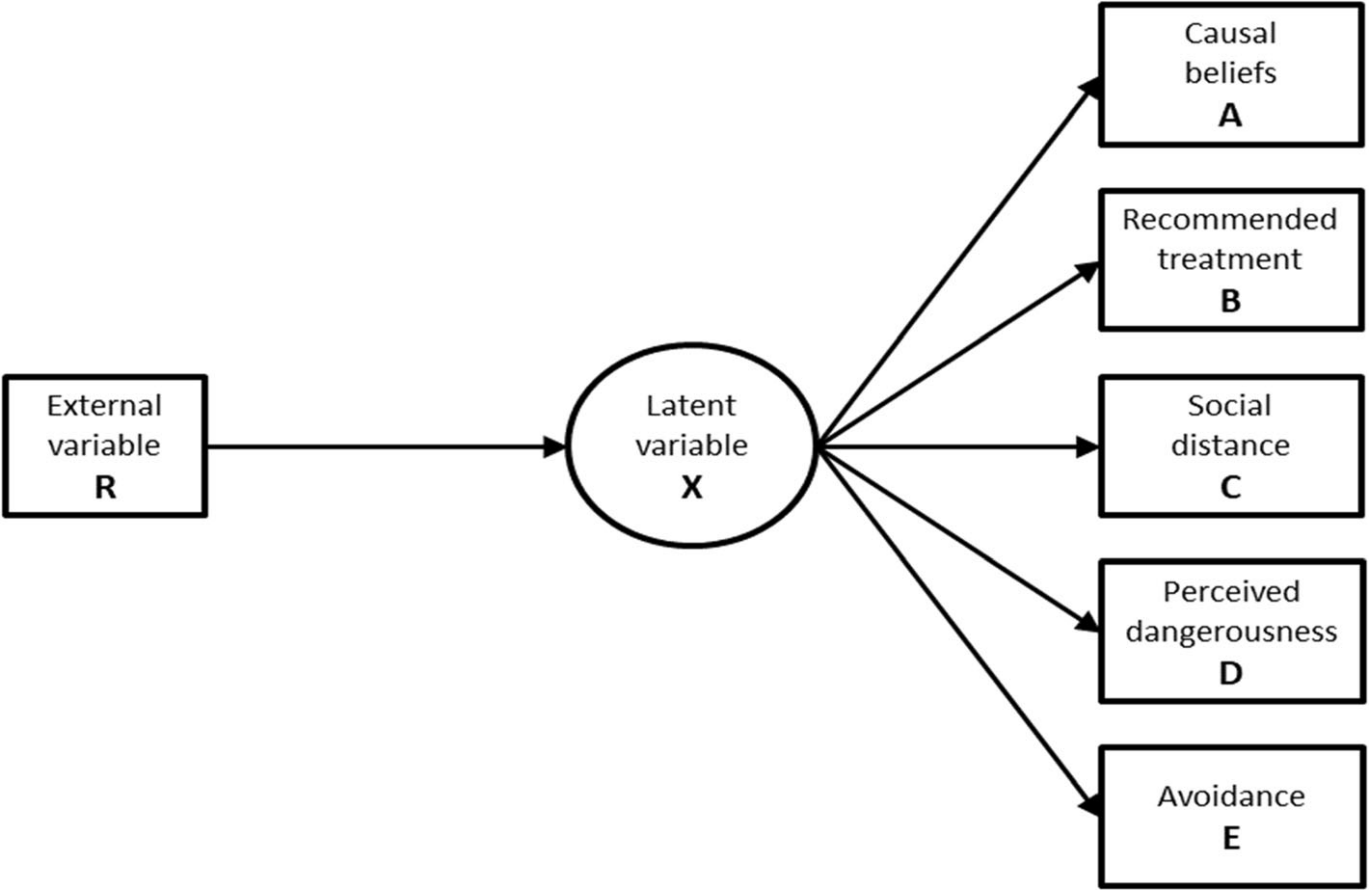
LCA conceptual model: one latent variable X, five manifest variables (A – “etiological beliefs”; B – “recommended treatment”; C – “social distance”, D – “dangerousness”; E – “avoidance”), and one external variable R (role)

The expected LCA model is depicted in Fig. 1, where R (roles) is the external variable and A (causal belief), B (eligible treatment), C (social distance), D (perceived dangerousness) and E (avoidance) are the manifest variable indicators of the latent variable X, schizophrenia. Bivariate interactions were also hypothesised between each manifest variable and the others. The model fitting the data was assessed by the Akaike Information Criterion (AIC) [67] and the Bayesian Information Criterion (BIC) [68]. The likelihood ratio statistics was also calculated [69]. The LCA analysis was performed with the LEM program [70].

## Results

### Respondents’ characteristics

Of the respondents, 157 were *psychiatric patients*, of whom 36.8% reported a diagnosis of mood spectrum disorder, 35.1% a diagnosis of psychosis spectrum disorder, 23.6% a diagnosis of anxiety spectrum disorder and 4.6% a diagnosis of personality spectrum disorder. Moreover, 35.1% of patients reported suffering mental health disorders for up to 10 years, 24.1% from 10 to 20 years and 40.5% for more than 20 years. In addition, 36.2% of this sample reported being in treatment by a psychiatrist only, while 63.6% reported being in treatment by both a psychiatrist and a psychotherapist. Finally, 86.8% of this sample reported taking medications regularly.

*Relatives* accounted for 135 of respondents and comprised different kinds of relatives: 16.3% were parents, 41.3% sons, 23.8% siblings, 12.5% spouses and 6.3% other kin. Moreover, 47.5% of the relatives reported to have a family member with a diagnosis of psychosis spectrum disorder, 28.7% a diagnosis of mood spectrum disorder, 13.8% a diagnosis of anxiety spectrum disorder and 10% a diagnosis of personality spectrum disorder. Moreover, 42.5% of participants reported that their family member had been suffering from a mental health disorder for up to 10 years, 18.8% from 10 to 20 years and 38.8% for more than 20 years. In addition, when the patients’ relatives were asked if they also undertook or had undertaken some kind of psychological or psychiatric treatment, 37.5% reported being in treatment by a psychiatrist only, while 62.5% reported being in treatment by both a psychiatrist and a psychotherapist. Finally, 78.8% of these participants reported taking medications regularly.

*Mental Health (MH) professionals* accounted for 102 of respondents and comprised several kinds of professionals: 57.8% of these respondents were psychiatric nurses, 16.7% psychiatrists, 14.4% psychologists, 5.5% social workers and 5.5% educators. Moreover, 5.5% of this group had worked with patients with mental health disorders for up to 3 years, 5.5% from 3 to 5 years, 35.5% from 5 to 10 years, 14.4% from 10 to 15 years and 38.8% for more than 15 years. In addition, 35.5% had asked for help from a mental health professional in his/her lifetime and 84.4% of the sample reported taking medication regularly.

*Psychology students* accounted for 183 of the respondents; in the majority, they were master’s degree students. Of the participants, 78.1% had contact with people suffering from mental health disorders. In greater detail, 16.9% of such a person was one of his/her parents, 21.3% was a relative, 14.2% was a friend, 9.3% was an acquaintance and 2.1% was a neighbour, while 36.1% referred to having experienced mental illness during his/her practice in clinical environments.

### Latent class analysis

#### Latent classes

Three LCAs with two, three or four classes were elaborated in order to find the best model fitting the data. As expected, the four LCAs identified the best model (L^2^ = 183.8, *df* = 172, *p* = .26; AIC = − 160.19, BIC = − 909.74), whereas the two- and three-class models presented unsatisfactory statistics, in particular the AIC and BIC indicators. The four model parameters revealed the peculiarities of each class, as shown in Table 2.

**Table 2:** LCA probability values for the external variable R and the manifest variables A, B, C, D, and E for each latent class X

Class X		Role				Causal belief			Treatment			Distance		Dangerousness		Avoidance	
		PA	PR	RE	ST	PSY	P/B	BIO	PSY	P/M	MED	low	high	low	high	low	high
1	.26	.30	**.41**	.29	.00	.11	.43*	.46*	.45*	.35	.20	.26	.74*	.42	.58*	.30	.70*
2	.27	.31	.14	**.42**	.13	.55*	.16	.29	.59*	.15	.26	.35	.65*	.62*	.38	.32	.68*
3	.19	**.37**	.05	.25	.33	.14	.41*	.45*	.41*	.09	.50*	.38	.62*	.81*	.19	.67*	.33
4	.28	.13	.08	.01	**.78**	.46*	.43*	.11	.10	.35	.54*	.61*	.39	.63*	.37	.73*	.27

Note: * latent class probability higher than the considered threshold (.35). *PA* patient, *PR* professional, *RE* relatives, *ST* student. *PSY* psychological, *P/B* psycho/biological, *BIO* biological, *P/M* psycho/medical, *MED* medical

**Fig. 2:**
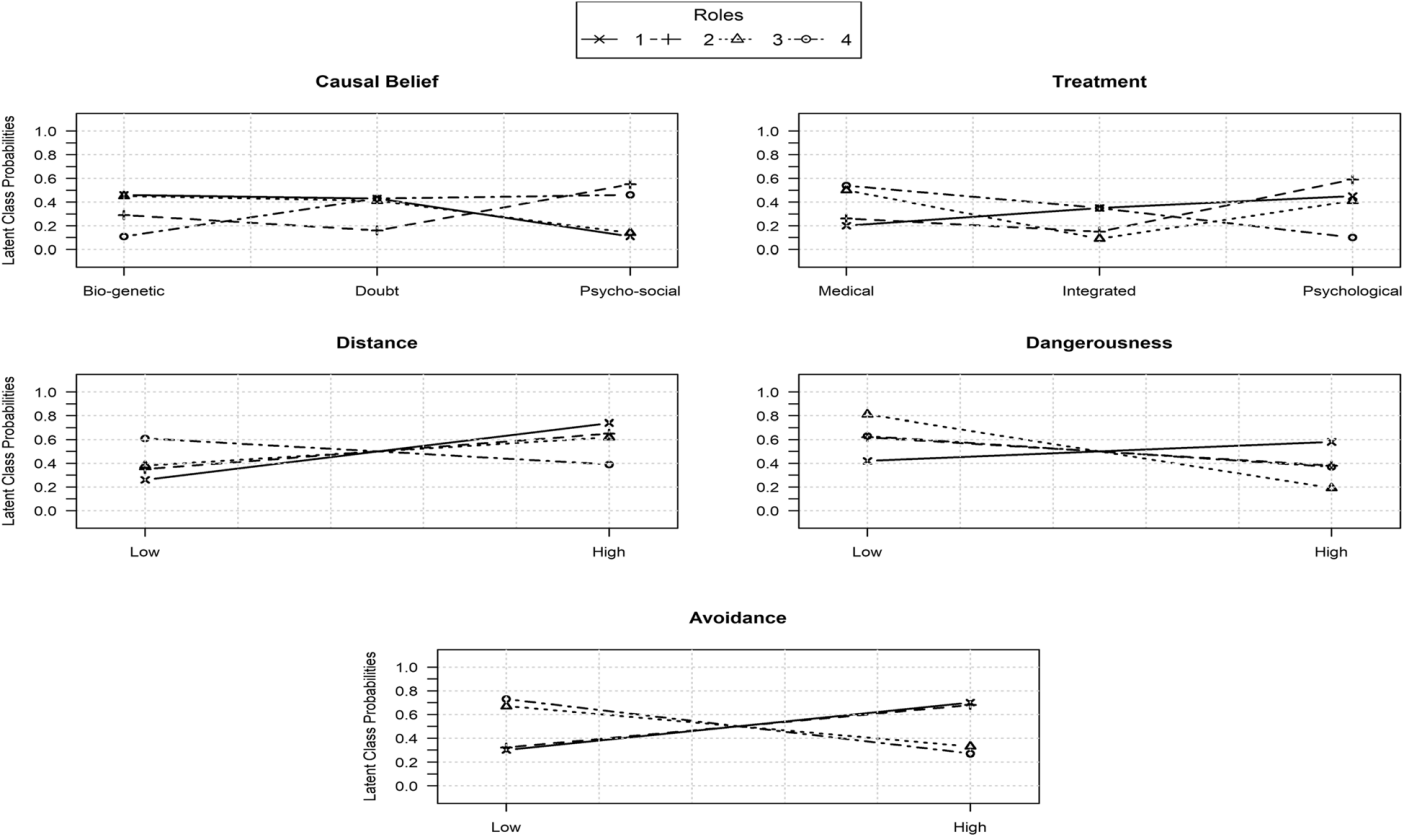
Comparison among four latent classes (MH Professionals 1; Relatives 2; Patients 3; Students 4) probability values in relation to manifest variables

Each class was associated with a probability value indicating its relevance. Each class was then denominated according to the role category with the highest probability in that class. In this study, probability .35 was considered as a threshold [50, 71] in order to denominate a class. To better delineate the position of each role in the context of the schizophrenia indicators (causal belief, treatment, distance, danger and avoidance), also probabilities slightly lower than .35 (between .29 and .35) associated with a class were taken into consideration for interpretation (see Table 2). Differences and analogies between roles are graphically presented in Fig. 2.

*Class 1* was associated with probability *p* = .26, and such a class was mainly represented by mental health professionals (41%), which was then denominated as professionals (PR). In this class, patients (PA) and relatives (RE) at a lower level, also showed relevant probabilities, respectively .30 and .29. Class 1 was characterised by high social distance, high dangerousness and high avoidance, which were associated with the following probability values, respectively: .74, .58 and .70. Furthermore, both biological causal beliefs (.46) and psychobiological ones (.43), together with psychological treatments (.45), were also endorsed by the respondents.

*Class 2* showed a probability of *p* = .27 and was mainly represented by relatives (42%), which were denominated as relatives (RE). In Class 2, causal belief and treatment probability values demonstrated a clear tendency towards a psychosocial causal belief (.55) and psychological treatments (.59); as far as schizophrenic characteristics are concerned, a desire for distance (.65), low perceived dangerousness (.62) and high public avoidance (.68) were also demonstrated.

*Class 3* had a probability of *p* = .19 and was represented by patients with a value of 37%; it was denominated as PA. Mixed results emerged as far as causal beliefs and treatments are concerned, namely, both biological (.45) and psychobiological causal beliefs (.41) and both medical (.50) and psychological treatments (.41) were endorsed. Also, high social distance (.62), low dangerousness (.81) and low public avoidance (.67) showed high probabilities.

*Class 4* was associated as a probability of *p* = .28; it was characterised by the students as respondents covering the 78% probability of belonging to that class. Class 4 was then denominated as students (ST). The majority of the respondents who were characterised as Class 4 were more likely to express low distance (.61) towards schizophrenia, low dangerousness (.63) and low avoidance (.73); they recommended medical treatments (.54) and endorsed with considerable probabilities both psychosocial (.46) and psychobiological causal beliefs (.43).

Figure 2 illustrates the position of each role, in the context of the LCA schizophrenia indicators structure, namely causal belief, treatment, distance, danger and avoidance.

*Causal belief –* Fig. 2 shows a very clear analogy between mental health professionals’ and patients’ preferences; both groups of participants expressed a clear preference for genetic/biological causes as the origin of schizophrenia, with probability values of .46 and .45, respectively. Such an opinion seemed to be in contrast with the opinions of relatives and students whose probabilities were, respectively, .29 and .11. Relatives and students believed in a psychosocial origin for schizophrenia with probabilities of .55 and .46, respectively, whereas mental health professionals and patients expressed their preferences for psychosocial causes with much lower probabilities, of .11 and .14, respectively. This is to underline that a certain analogy also exists between mental health professionals and patients concerning the psychobiological response category to be medium with probabilities of .43 and .41, respectively. This category is also endorsed by students with a probability of .43.

*Treatment –* Fig. 2 illustrates a clear contrast in the opinions expressed by medical health professionals and students, namely, medical health professionals recommended psychological treatments with a probability of .45, whereas students gave their preference to medical treatments (.54). A certain analogy is evident between these two groups of respondents in relation to the medium category; they both endorsed the category of an integrated medical/psychological treatment with a probability of .35. Families and patients showed a certain analogy in choosing psychological treatments with the probabilities of .59 and .41, respectively, but also some differences since patients also expressed a preference for medical treatments with a higher probability (.50). Furthermore, professionals and relatives showed some similarities in endorsing psychological treatments with probabilities of .45 and .59, respectively. This result seemed to be in contrast with patents’ (.50) and students’ (.54) preferences for medical treatments. Mental health professionals and students showed some similarities when endorsing an integrated psycho/medical treatment, whereas patients also manifested a preference for psychological treatments.

*Distance* – Fig. 2 presents the evidence for some analogies in the opinions of professionals, relatives and patients. The majority of those participants expressed a preference for a high distance from schizophrenia with the probabilities of .74, .65 and .62. Students, in contrast, endorsed the low distance category with a probability of .61.

*Danger –* Fig. 2 shows the very clear opinion expressed by patients who endorsed low dangerousness as far as schizophrenia is concerned with a probability of .81, in line with patients’ opinions, but with lower probabilities are relatives and students, respectively, with values of .62 and .63. The majority of professionals disagreed, with a probability of .58.

*Avoidance –* Fig. 2 illustrates a clear analogy between mental health professionals’ and relatives’ opinions. They both endorse the category of high avoidance with the considerable probability values of .70 and .68, respectively. On the contrary, patients and students in agreement expressed a preference for the category of low avoidance with probabilities of .67 and .73, respectively.

### Log-linear analysis

#### Association between LCA variables

LCA results demonstrated the consistency of the expected LCA model, namely, the latent variable X demonstrated interpretable relations between participants’ roles and their thinking and preferences towards schizophrenia. To further demonstrate and confirm such results, the interactions between the latent variable X and each variable, namely roles (R), causal belief (A), treatment (B), distance (C), dangerousness (D) and avoidance (E), were tested by means of bivariate log-linear analyses. The interactions between each manifest and latent variable and between manifest variables were also analysed. The Wald test statistics were significant (*p* < .001) for all log-linear analyses, except for CxE and DxE interactions. These results showed that participants’ roles (R) are strongly associated with the latent variable X, and variables A, B, C, D and E were demonstrated to be good indicators of X. Standardised log-linear parameters for the interactions between the latent variable and each manifest variable were significant (*p* < .001), confirming in large part the LCA results. Furthermore, considering all the participants in a single group, without distinguishing the roles, significant positive parameters (*p* < .001) were associated with all the bivariate interactions between manifest variables except for cross-tabulated variables CxE and DxE. In particular, positive significant parameters confirmed the relations between psychosocial causal belief and psychological treatment and between biological causal belief and medical treatment (AxB). The biological causal belief was positively associated with low distance (AxC), low dangerousness (AxD) and low avoidance (AxE). Biological treatment was also positively associated with low distance (BxC), low dangerousness (BxD) and low avoidance (BxE). Positive significant parameters were also associated with the interactions between low distance and low dangerousness (CxD).

## Discussion

In this study, two main problems concerning thinking and beliefs towards schizophrenia were analysed: first, whether schizophrenia indicators, such as causal belief, eligible treatment, social distance, dangerousness and avoidance, might be represented by an underlying latent variable, and, secondly, whether different groups of individuals involved with schizophrenia with different roles could be considered moderators of the same latent variable. Both problems found satisfactory solutions on the basis of an LCA, which allowed demonstrating a structure characterised by significant relations among all variables at a latent level. As hypothesised, the results demonstrated a latent structure characterised by the relevance of the four manifest variables as indicators of schizophrenia and the effect of the external variables, namely the four roles of participants, on the latent structure. Such results allowed interpreting four latent classes, each one describing a profile typical of a specific role. *Class 1* was characterised by mental health professionals with a probability of .41; they preferably endorsed a biological origin for schizophrenia, but with a slightly lower probability, and they also indicated psychobiological causes. As regards treatment, they preferred a psychological approach. Concerning stigmatising attitudes, all three aspects analysed were considered highly present, namely dangerousness, social distance, in terms of being personally involved in their private lives, and public avoidance. *Class 2* was mainly represented by relatives with a probability of .42. Differently from mental health professionals, relatives believed in a psychological origin of schizophrenia, excluding biological causes and confirming Magliano and colleagues’ results, where it was demonstrated that relatives more frequently adhere to a psychological model; similarly to professionals’ thinking, relatives endorsed psychological treatments [32, 72]. According to Magliano and colleagues’ interpretation, the low importance given to biological causal beliefs by relatives is probably partially due to strategies adopted to cope with feelings of guilt associated with a hereditary transmission of the illness [32]. Relatives did not think that persons with schizophrenia are dangerous and they believed that, in general, people do not avoid them, but when asked about the social distance, the prevalent opinion was that being too close to a person with schizophrenia is not desirable. *Class 3* was characteristic of patients’ beliefs with a probability of .33. Their opinions about the origin of the disorder and about the possible treatments were not well defined; they indicated both biological and psychobiological causes and a mixed approach of medical and psycho-medical treatments. They considered the person depicted in the vignette to be not dangerous, but they expressed both a high desire for personal distance from that person and thought that also people, in general, would avoid him/her leading to a sense of loneliness [34]. *Class 4* was characterised by students with a probability of .78 for belonging to that class. The majority did not express a desire for distance from persons with schizophrenia; they also did not consider them dangerous and they believed that, in general, people do not avoid them. They recommended medical treatments and endorsed both a psychosocial and a psychobiological causal belief.

These findings confirmed in part previous results obtained in the Italian environment; in particular, some incongruent aspects concerning schizophrenia indicators were confirmed in this study [4, 11]. In some cases, social distance, dangerousness and avoidance seemed to be in opposition: relatives thought that schizophrenia is not dangerous and that in general people do not avoid individuals with schizophrenia, but at the same time, they thought that living personally too close to those persons was not desirable. An interpretation might be that the incongruence of the participants’ feelings as regards distance and avoidance is only apparent, since they are two different constructs as they are presented to the participants. In other words, relatives who live very close to a schizophrenic person, know very well how complicated it is, in terms of affectivity and responsibilities [73]. Another contrast is found when analysing patients’ responses; they considered the person depicted in the vignette as not dangerous, but they thought that they would not like to have a close involvement with him/her and that also, other persons would avoid him/her. This result seemed to demonstrate that the reason for refusing a mentally ill person is not a feeling of being in danger; rather, a personal relationship, such as either being a close friend, a colleague or a neighbour, seemed to be the cause of the refusal. Considering the schizophrenia, there was an analogy between MH professionals’ and patients’ thoughts; they both endorsed a biological origin, plus they also indicated a significant probability of psychobiological causes. Also, students expressed a mixed choice as regards causes of schizophrenia, with a higher probability they endorsed a psychosocial origin, but they also indicated a psychobiological one. Only relatives gave a clear answer; they believed that the cause is psychosocial. As far as eligible treatment is concerned, both MH professionals and relatives indicated a preference for psychological interventions, whereas patients and students indicated a medical approach, while patients also showed their preference for an integrated approach of medical and psychological cures.

Comparing this study’s results with those of Tarakita et al. (2019), some analogies and differences can be demonstrated. Those authors found that biological conceptions were the most predominant causes considered by professionals, while psychosocial stressors were endorsed by patients and their relatives. Furthermore, as far as danger and risk are concerned, conceptions of risk among professionals were significantly higher than those among patients and their families. As Tarakita explains, medical staff may acquire various causal beliefs about schizophrenia from textbooks or from their experiences with schizophrenic patients, while patients and their families tend to form their specific causal beliefs of schizophrenia through their own experiences. As far as the relation between treatment and schizophrenia causes is concerned, differently from this study’s results, Tarakita’s interpretation of their findings demonstrates that professionals might think that biological treatment is efficient in treating schizophrenia because their etiological beliefs regarding schizophrenia are mainly related to biological factors. However, patients and their families hope that psychotherapy focuses on psychosocial factors in addition to pharmacotherapy.

In order to further clarify the possibility of overcoming the limitations of this study, first of all, other types of mental disorders should be taken into consideration by comparing them with schizophrenia. Namely, a latent structure might be hypothesised where different groups of participants and different mental disorders, such as anxiety bulimia, addiction and depression, should interact within a complex latent structure [4]. Furthermore, other schizophrenia indicators and stereotypes might be included, in order to better describe the stigmatisation problems regarding mental illness. Furthermore, a strictly medical staff group should be introduced for comparison with a more generally defined mental health professional group [31].

A limitation of the study may be related to the different durations of contact with the mental disease between and within the groups (e.g., wide duration of illness in psychiatric patients). However, this variability might be more representative of reality, thus leading to greater ecological validity of the results, as in the real context there are strong variations regarding contact with mental illness. Future research could fill this lack by studying how different durations of contact with mental illness can affect the components of the stigma.

## Conclusions

In general, the findings of this study are consistent with the evidence that people’s knowledge and thinking about mental disorders and in particular schizophrenia, are significantly related to the involvement that people have with the mental disorder. Education, experience and direct contact with mental illness have a relation with causal and treatment beliefs and stigmatising attitudes about schizophrenia.

## Data Availability

The datasets used and/or analyzed during the current study are available from the corresponding author on reasonable request.

## Abbreviations

LCA: Latent Class Analysis
AIC: Akaike Information Criterion
BIC: Bayesian Information Criterion
